# Impact of sarcopenia on the future liver remnant growth after portal vein embolization and associating liver partition and portal vein ligation for staged hepatectomy in patients with liver cancer: A systematic review

**DOI:** 10.3389/fonc.2022.1064785

**Published:** 2022-11-24

**Authors:** Qiang Wang, Anrong Wang, Zhen Li, Ernesto Sparrelid, Torkel B. Brismar

**Affiliations:** ^1^ Division of Medical Imaging and Technology, Department of Clinical Science, Intervention and Technology (CLINTEC), Karolinska Institutet, Stockholm, Sweden; ^2^ Department of Radiology, Karolinska University Hospital Huddinge, Stockholm, Sweden; ^3^ Department of Vascular Surgery, The First Affiliated Hospital of Chongqing Medical University, Chongqing, China; ^4^ Department of Interventional Therapy, People’s Hospital of Dianjiang County, Chongqing, China; ^5^ Department of Hepatobiliary Surgery, People’s Hospital of Dianjiang County, Chongqing, China; ^6^ Division of Surgery, Department of Clinical Science, Intervention and Technology (CLINTEC), Karolinska Institutet, Karolinska University Hospital, Stockholm, Sweden

**Keywords:** sarcopenia, body composition, liver growth, portal vein embolization, ALPPS, liver cancer

## Abstract

**Purpose:**

The impact of sarcopenia on the future liver remnant (FLR) growth after portal vein occlusion, including portal vein embolization (PVE) and associating liver partition and portal vein ligation for staged hepatectomy (ALPPS) has gained increasing interest. This systematic review aimed to explore whether sarcopenia was associated with insufficient FLR growth after PVE/ALPPS stage-1.

**Methods:**

A systematic literature search was performed in PubMed, Embase, Web of Science, and Cochrane Library up to 05 July 2022. Studies evaluating the influence of sarcopenia on FLR growth after PVE/ALPPS stage-1 in patients with liver cancer were included. A predefined table was used to extract information including the study and patient characteristics, sarcopenia measurement, FLR growth, post-treatment complications and post-hepatectomy liver failure, resection rate. Research quality was evaluated by the Newcastle-Ottawa Scale.

**Results:**

Five studies consisting of 609 patients were included in this study, with a sample size ranging from 42 to 306 (median: 90) patients. Only one study was multicenter research. The incidence of sarcopenia differed from 40% to 67% (median: 63%). Skeletal muscle index based on pretreatment computed tomography was the commonly used parameter for sarcopenia evaluation. All included studies showed that sarcopenia impaired the FLR growth after PVE/ALPPS stage-1. However, the association between sarcopenia and post-treatment complications, post-hepatectomy liver failure, and resection rate remains unclear. All studies showed moderate-to-high quality.

**Conclusions:**

Sarcopenia seems to be prevalent in patients undergoing PVE/ALPPS and may be a risk factor for impaired liver growth after PVE/ALPPS stage-1 according to currently limited evidence.

**Systematic review registration:**

https://inplasy.com/, identifier INPLASY202280038.

## Introduction

Liver resection remains a mainstay treatment for patients with primary or secondary liver cancer (for instance, hepatocellular carcinoma or colorectal liver metastases) with curative intent (1). However, many patients have been at an advanced stage at first diagnosis, and only 15-25% of patients are indicative of liver resection (2). For those patients who are not eligible for surgery, a majority of them are due to the limited future liver remnant (FLR), which is the remaining part of the liver after liver resection, and it serves as a key determinant for extended liver resection (3). FLR has to be sufficient to maintain normal physiologic function after liver resection, otherwise a lethal complication, post-hepatectomy liver failure will occur (4). To prevent the occurrence of liver failure after liver resection, the FLR volume limit should be > 20% of the total liver in a normal liver, > 30% in the abnormal liver (such as steatosis or post-chemotherapy), and at least 40% in the cirrhotic liver (5).

In clinical practice, many strategies have been proposed to increase the size of the FLR volume before extended liver resection. Portal vein embolization (PVE) is the commonly used technique and was first introduced by Masatoshi Makuuchi in the 1980s (6). At PVE, the branch of the portal vein leading the blood to the diseased lobes of the liver is occluded interventionally by using sponges or metal coils. By this interruption of the blood flow, the un-embolized lobes (i.e. the FLR) will be exposed to all the portal venous blood flow. This increase in flow, including exposure to nutrients, toxins, and oxygen triggers liver growth (7). Most often, after waiting for several weeks, a sufficient growth of the FLR volume has occurred and a radical liver resection can be performed safely. PVE is still the standard procedure before extended liver resection when the FLR volume is estimated to be insufficient (8). Typically, an FLR growth of 12-38% can be observed within 4-8 weeks after PVE (9). However, during the waiting period, approximately 20-40% of patients cannot proceed to hepatic resection due to insufficient liver growth or tumor progression (9, 10). Furthermore, patients with poor liver growth after PVE also have an increased risk of post-intervention complications (11).

Hepatobiliary surgeons have been committed to developing an improved method to overcome the above-mentioned limitations of PVE. In recent years, a novel strategy, called associating liver partition and portal vein ligation for staged hepatectomy (ALPPS) has been proposed (12, 13). It contains two steps: in the first step, after the branch of the portal vein to the diseased lobes has been ligated (PVL, rather similar to the PVE procedure), the liver parenchyma is transected between the ligated part and the unligated part (i.e. the FLR). Once the FLR volume has increased sufficiently, liver resection can be performed to remove the liver tumor in the second step (12, 14). Interestingly, ALPPS can trigger an accelerated FLR growth in a shorter time than PVE, with a 40-80% FLR increase in only 6-9 days (12–15). However, ALPPS has high perioperative morbidity and mortality due to major surgical trauma, and the FLR growth varies among patients after ALPPS stage-1 (14). Despite surgically successful ALPPS stage-1, not all patients can complete the liver resection (14).

It is therefore of clinical importance to identify pretreatment factors that indicate a risk for insufficient FLR growth, which might allow optimizing treatment management of patients with liver cancer. Many clinical variables have been identified to be predictive for insufficient liver growth after PVE/ALPPS stage-1, for example, age, body mass index, and the diseased liver parenchyma (16). Among those, body composition is drawing increasing attention and has been assumed to be a treatable, prognostic factor in several hepatopancreatobiliary cancers after surgery (17–19). Sarcopenia is characterized by a progressive, generalized loss of skeletal muscle mass and function with aging (20). Previous studies have demonstrated sarcopenia to be associated with poor overall survival, early tumor recurrence, prolonged intensive care unit, and hospital stay after liver resection (21, 22). In recent years the influence of sarcopenia on the FLR growth after PVE/ALPPS stage-1 has been studied. However, no research systematically summarizes the results of these studies to date. This study aimed to provide such a systematic review.

## Methods and materials

The research protocol was prospectively registered at the public platform International Platform of Registered Systematic Review and Meta-analysis Protocols (https://inplasy.com/) with registration number INPLASY202280038. This study was carried out in accordance with the guidance of the Preferred Reporting Items for a Systematic Review and Meta-analysis (PRISMA) (23). The PRISMA checklist can be found in **Supplementary Table S1**.

### Literature search and study selection

A systematic literature search was performed at four public databases: PubMed, Embase, Web of Science, and Cochrane Library and was last updated on 5 July 2022. A search strategy combining Medical Subject Headings (MeSH) terms and text words were adopted. The keywords for literature search included “sarcopenia”, “body composition”, “portal vein embolization”, “portal vein ligation”, “portal vein occlusion”, “associating liver partition and portal vein ligation for staged hepatectomy”. The detailed search queries are provided in **Supplementary Table S2**.

Records satisfying the following criteria were regarded as eligible: 1) prospective or retrospective observational studies; 2) patients with liver cancer who underwent PVE/portal vein ligation or ALPPS to induce FLR growth before liver resection; 3) FLR growth as the main outcome or one of the outcomes; 4) at least one index for sarcopenia or body composition assessment involved. Studies would be excluded if they were: 1) in the forms of narrative review, letter, reference abstract, editorial, and case report; 2) animal research.

The process of study selection was carried out by two researchers (Q.W & A.W) independently by reading the title and abstract first to screen potentially ineligible studies. After that, the full text of the screened studies was obtained to further check their eligibility in consensus. Previous reviews and the reference list of the eligible studies were also manually retrieved to detect potential eligible studies.

### Data extraction and research quality evaluation

The same researchers (Q.W & A.W) independently extracted the data from the included studies and assessed the research quality. The extracted information included: study characteristics (first author, publication year, country, study design, single or multiple center studies, and sample size), patient characteristics (age, gender ratio, the procedure involved, indication, and whether also segment IV was embolized), sarcopenia related information (modality used, body composition measurement, body composition parameters, sarcopenia definition, and the incidence of sarcopenia), FLR growth (degree of hypertrophy and kinetic growth rate), independent risk factors for poor FLR growth, complications/post-hepatectomy liver failure, the liver resection rate, and the main finding of the study.

Research quality and risk of bias of the cohort or case-control studies were evaluated by using the Newcastle-Ottawa Scale tool, which is a validated and easy-to-use scale containing eight items within three domains (selection of study groups, comparability of groups, and ascertainment of exposure/outcomes) (24). The maximum score of this tool is 9, with 7-9 indicating high quality, 4-6 moderate quality, and 0-3 low quality (24). Research quality and risk of bias of the cross-sectional study were assessed by applying the Agency for Healthcare Research and Quality tool, which contains an 11-item checklist (25). The quality grades were defined as follows: 8-11 (high quality), 4-7 (moderate quality), and 0-3 (low quality). Any disagreement in data extraction and research quality appraisal between the two researchers was solved by discussion or by consulting a senior researcher (T.B.B).

## Results

### Study characteristics and research quality assessment

**Figure 1:**
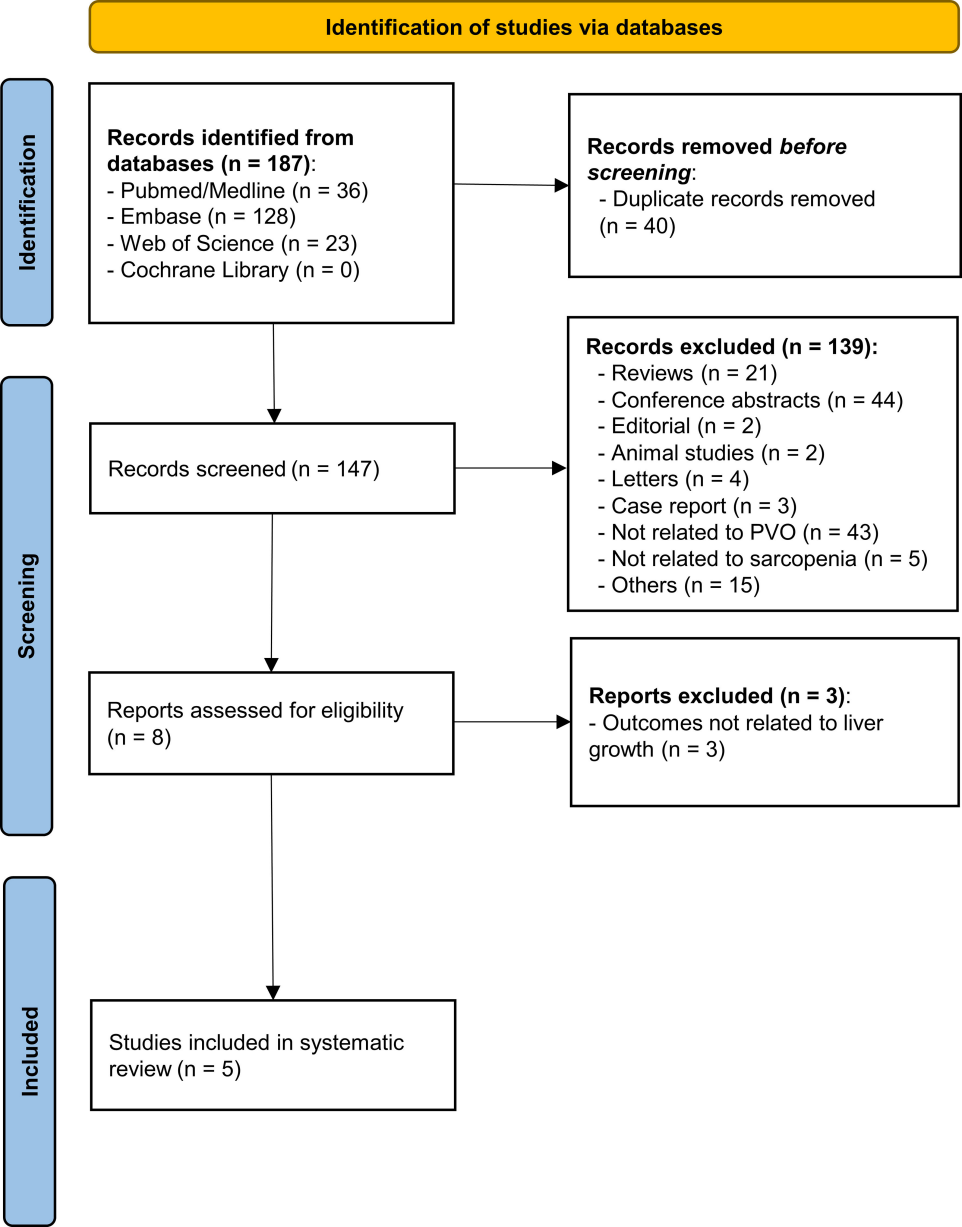
The study selection process of this study. A total of 187 records were initially identified in the four public databases. After the removal of 182 ineligible publications *via* reading the title, abstract, and full text, five studies were finally included in this systematic review. PVO, portal vein occlusion.

Systematic literature searching initially yielded 187 records from the four electronic databases. After the removal of ineligible studies (duplications (40), inappropriate form of research (71), animal research/case report (5), studies not related to portal vein occlusion or sarcopenia (63), and no liver growth indices available (3)), five studies remained for inclusion in this systematic review (26–30). The process of study selection is shown in **Figure 1** and **Supplementary Table S3**.

The five studies were published between January 2020 and May 2022 and were all retrospectively designed. A total of 609 patients were evaluated, with a sample size ranging from 42 to 306 (median: 90) patients. Only one study was carried out at multiple medical centers, which were located in several European countries (29). Only one study was conducted in an Asian country (28). The Newcastle-Ottawa Scale score of the four cohort/case-control studies varied from 6 to 9 (median: 6.5) (moderate-to-high quality), while one cross-sectional study was assigned an Agency for Healthcare Research and Quality score of 6 (moderate quality) (**Table 1**).

**Table 1:** Study and patient characteristics.

Study ID	Publication year	Country	Study design	Single/ multiple center	Sample size	Age (years)	Gender(M/F)	Procedure	Indication	Segment IV embolization	NOS score
**Schulze-Hagen[** 26 **]**	2020	Germany	Retrospective, cross-sectional study	Single	42	63	32/10	PVE	CRLM	No	6†
**Denbo[** 27 **]**	2020	USA	Retrospective, cohort study	Single	45	58	31/14	PVE	CRLM	No	6
**Yao[** 28 **]**	2021	Japan	Retrospective, cohort study	Single	126	68	80/46	PVE	CCA (48%), HCC (15%), Metastatic tumor (29%), GBC (8%)	Unclear	6
**Heil[** 29 **]**	2021	Seven European countries	Retrospective, cohort study	Multiple	306	64/62#	183/123	PVE	CRLM (56%), HCC (7%), IHCC (12%), PHCC (15%), GBC(6%),others(4%)	37 (12%) cases	9
**Reese[** 30 **]**	2022	Germany	Retrospective, case-control study	Single	90	61/56##	65/25	ALPPS	CRLM(69%), other metastasis(11%), HCC(9%), IHCC (8%), PHCC(2%), GBC(1%)	Unclear	7

# the sarcopenic vs non-sarcopenic groups; ## Low vs high kinetic liver growth groups; † scored by Agency for Healthcare Research and Quality tool; ALPPS, associating liver partition and portal vein ligation for staged hepatectomy; CCA cholangiocarcinoma; CRLM, colorectal liver metastasis; GBC, gallbladder carcinoma; HCC, hepatocellular carcinoma; IHCC: intrahepatic cholangiocarcinoma; NOS, the Newcastle-Ottawa Scale tool; PHCC: perihilar cholangiocarcinoma; PVE, portal vein embolization.

### Patient characteristics

The average age of the included patients was between 56 and 68 years. A predominance of males was found in all studies (in total, 391/609 = 64%), which is typical of the diseases involved. Two studies exclusively focused on patients with colorectal liver metastases (26, 27), while the patients in the other three studies had varying indications. Four studies evaluated the impact of sarcopenia in patients undergoing PVE while the remaining one evaluated patients with ALPPS (30) (**Table 1**).

### Skeletal muscle measurement and definition of sarcopenia

All body composition analyses were based on pretreatment computed tomography (CT) images: two studies stated the CT image phase used (one without contrast media and the other on images obtained in the portal venous phase) (26, 28). A slice thickness of 5 mm was reported in three studies (26, 29, 30), while slice thickness was not reported in two (27, 28). All studies measured the skeletal muscle area (at the level of the third lumber vertebra), which was converted into skeletal muscle index by being divided by squared height (m^2^). Three studies adopted the skeletal muscle index to define sarcopenia (27–29). All three studies used the same threshold levels, including the one from Japan; sarcopenia was defined by a threshold of skeletal muscle index < 41 cm^2^/m^2^ in women, while in men two thresholds were used depending on the body mass index; at body mass index < 25 kg/m^2^ a skeletal muscle index < 43 cm^2^/m^2^ defined sarcopenia, while the threshold was skeletal muscle index < 53 cm^2^/m^2^ when body mass index was > 25 kg/m^2^ (27–29). The incidence of sarcopenia in the three studies ranged from 40% to 67% (median: 63%) (27–29).

One study applied the parameter “muscularity” to comprehensively evaluate muscle quantity and quality (28). This parameter combines skeletal muscle index and intramuscular adipose tissue content to represent both skeletal muscle quantity and quality. Another study, which was an early study with a limited sample size, did not provide their definition of sarcopenia but explored the correlation between muscle indices and liver growth (26). With a case-control design, the ALPPS study dichotomized patients using a threshold of the kinetic growth rate of 7%/week (30). The difference in skeletal muscle index between the two groups was then compared. Detailed information about sarcopenia measurement can be found in **Table 2**.

**Table 2:** Body composition measurement and sarcopenia definition.

Study ID	Modality for body composition measurement	Body composition measurement	Muscle quantity Parameter(s)	Muscle quality parameter	Other parameter(s)	Sarcopenia definition	Sarcopenia cases (incidence)
**Schulze-Hagen[** 26 **]**	5 mm slice, portal venous CT images	Skeletal muscle area at L3 (SMI, SMA); the largest psoas muscle diameter (PMCS); automatic machine learning algorithm (PMV)	PMV, PMCS, SMI, SMA	No	NA	NA	NA
**Denbo[** 27 **]**	CT images	Skeletal muscle area, visceral adipose area, and subcutaneous adipose area at L3	SMI	No	VAI, SAI	SMI < 41 cm^2^/m^2^ (women); SMI < 43 cm^2^/m^2^ (men with BMI of < 25 kg/m^2^), and < 53 cm^2^/m^2^ (men with BMI > 25 kg/m^2^)	18 (40%)
**Yao[** 28 **]**	Plain CT images	Skeletal muscle area, visceral adipose area, and subcutaneous adipose area at L3	SMI	IMAC	Visceral-to-subcutaneous adipose tissue area ratio	SMI: 41 cm^2^/m^2^ (women); 43 cm^2^/m^2^(men with BMI < 25 kg/m^2^), and 53 cm^2^/m^2^ (men with BMI > 25 kg/m^2^). IMAC: - 0.229 (women) and - 0.358 (men).	85 (67%)
**Heil[** 29 **]**	5 mm slice CT images	Skeletal muscle area, visceral adipose area, and subcutaneous adipose area at L3	SMA, SMI	No	Subcutaneous adipose area, Visceral adipose area, SAI, VAI	SMI < 41 cm^2^/m^2^ (women); SMI < 43 cm^2^/m^2^ (men with BMI of < 25 kg/m^2^), and < 53 cm^2^/m^2^ (men with BMI > 25 kg/m^2^)	194 (63%)
**Reese[** 30 **]**	5 mm slice CT images	Area of the psoas major muscles at L3	SMI	NA	NA	NA	NA

BMI, body mass index; CT, computed tomography; IMAC, intramuscular adipose tissue content; L3, the 3rd lumbar vertebra; NA, not available/applicable; PMV, psoas muscle volume; PMCS, psoas muscle cross-sectional area; SAI, subcutaneous adipose index; SMA, skeletal muscle area; SMI, skeletal muscle index; VAI, Visceral adipose index.

### Liver growth rate

The degree of hypertrophy and kinetic growth rate of the FLR are two parameters commonly used for the assessment of liver growth after PVE/ALPPS stage-1. Two studies reported liver growth in the whole cohort, with a degree of hypertrophy of 8.9% and 9.5% respectively (26, 28). In three studies, the degree of hypertrophy in the sarcopenia and non-sarcopenia groups was evaluated, with a range of 8.0-8.3% and 10.8-15.2%, respectively (27–29). In the ALPPS research which dichotomized patients into low and high kinetic growth rate groups by a kinetic growth rate cutoff value of 7.0%/week, a degree of hypertrophy of 11% and 18% was observed in the two groups respectively (30) (**Table 3**). Compared with a kinetic growth rate of 2.6-4.0%/week in the non-sarcopenia group, the sarcopenia group demonstrated a significantly lower kinetic growth rate of 2.0%/week in two studies (27, 29). One study reported an overall kinetic growth rate of 3.6%/week for the whole study cohort (26).

**Table 3:** Future liver remnant growth, post-treatment complication rate and post-hepatectomy liver failure.

Study ID	Degree of hypertrophy of FLR	KGR of FLR	Independentrisk factorsfor poor liver growth	Complication rate/PHLF incidence	Surgical resection rate	Main findings
**Schulze-Hagen[** 26 **]**	8.9% (overall)	3.6%/week (overall)	NA	NA	NA	Psoas muscle volume and PMCS positively correlates with KGR of FLR after PVE
**Denbo[** 27 **]**	8.3% vs 15.2%*	2.0 vs 4.0%/week*	NA	NA	NA	Sarcopenia and related body composition indices are strongly associated with impaired liver growth after PVE
**Yao[** 28 **]**	9.5% (overall) 8.2% vs 10.8%*	NA	Initial FLR, total bilirubin, muscularity/IMAC^#^	52% vs 41% (N.S) for major complication^##^; 38% vs 17%* for PHLF grade B	83% (overall)	Low muscularity leads to poor liver hypertrophy after PVE and is also a predictor of PHLF
**Heil[** 29 **]**	8% vs 11%*	2.0 vs 2.6%/week*	Sarcopenia, initial FLR	31% vs 33% (N.S) for major complication; 16% vs 22% (N.S) for PHLF	73% (overall); 66% vs 87%*	Sarcopenia is associated with reduced KGR and resectability in patients undergoing PVE
**Reese[** 30 **]**	11% vs 18%^†^	Cut-off value: 7%/week	Body mass index, skeletal muscle index	20% vs 7% (N.S) for PHLF after ALPPS stage-1; 31% vs 7%* for PHLF after ALPPS stage-2^†^	87% (overall); 84% vs 93%(N.S)^†^	Low sarcopenia muscle index and a high body mass index correlate with impaired liver regeneration and increased liver dysfunction after ALPPS

Data comparison is presented as the **sarcopenia** versus **non-sarcopenia** groups, unless otherwise specified. * statistically significant; # according to two multivarible logistic regression models; ## by the Clavien–Dindo grading system; † low vs high KGR groups according to a cutoff value of 7%/week. ALPPS, associating liver partition and portal vein ligation for staged hepatectomy; FLR, future liver remnant; IMAC, intramuscular adipose tissue content; KGR, kinetic growth rate; PHLF, post-hepatectomy liver failure; PMCS, psoas muscle cross-sectional area; PVE, portal vein embolization; NA, not available/applicable; N.S, not significant.

In the three studies that performed multivariable logistic regression analysis, all identified sarcopenia as an independent factor for poor FLR growth (28–30). The other independent variables detected were initial FLR volume, total bilirubin level, and body mass index. All studies concluded that sarcopenia was associated with poor FLR growth after PVE/ALPPS stage-1 (**Table 3**).

### Post-treatment complications, post-hepatectomy liver failure, and resection rate

Two studies reported complications after PVE intervention, with a major complication (≥ III Clavien-Dindo classification) of 52% and 31% in the sarcopenia group versus 41% and 33% in the non-sarcopenia group respectively (both statistically non-significant) (28, 29). The incidence of post-hepatectomy liver failure was reported in two PVE studies and one ALPPS study. An opposite result was observed in the two PVE studies where the incidence of postoperative liver failure was 38% and 16% in the sarcopenia group versus 17% and 22% in the non-sarcopenia group respectively (one significant while the other not) (28, 29). The ALPPS study reported an incidence of post-hepatectomy liver failure of 20% and 7% after ALPPS stage-1 in the low and high kinetic growth rate groups respectively, but also here the difference was not statistically significant (30). There was a significant difference of the post-hepatectomy liver failure incidence between the low and high kinetic growth rate groups after ALPPS stage-2 (i.e. liver resection) with an incidence of 31% and 7%, respectively (p < 0.05) (30).

Overall resection rate was reported to be 83% and 73% respectively in two of the PVE studies (28, 29) and 87% in the ALPPS study (30). One study reported a significantly lower resection rate after PVE in the sarcopenia group, compared with the non-sarcopenia group (66% vs 87%) (29). Interestingly, as a study evaluated factors that might affect liver growth after PVE, one study excluded patients with insufficient FLR growth after PVE and only included patients who proceed to liver resection (28). In that study, 26 patients did not undergo liver resection. In the ALPPS study, the resection rate was 84% in the low kinetic growth rate group, but that was not statistically significantly less than the 93% resection rate in the high kinetic growth rate group (30). Detailed information can be found in **Table 3**.

## Discussion

The present study systematically reviews the association between skeletal muscle loss and FLR growth after PVE/ALPPS stage-1. A high incidence of sarcopenia among patients undergoing PVE was observed, and sarcopenia was associated with impaired FLR growth after PVE/ALPPS stage-1. However, its relationship with post-treatment complication rate, post-hepatectomy liver failure as well as surgical resection rate remains unclear.

The median incidence of sarcopenia among patients undergoing PVE in the included studies was 63%, which was higher than the reported incidence in patients with colorectal liver metastases (17-26%) (18, 31) or hepatocellular carcinoma (30-54%) (18), the two most common indications for PVE/ALPPS. Generally, the incidence of sarcopenia in patients with cancer has a wide variation due to different tumor types, tumor stages, measuring methods, and indices and criteria used (32). In the case of PVE/ALPPS, the indications usually vary among centers, which may also contribute to a varying and higher incidence of sarcopenia. Another explanation for the high observed incidence of sarcopenia is that the patients requiring PVE/ALPPS often have a chronically diseased liver such as liver cirrhosis (33) or have experienced several cycles of chemotherapy (e.g. neoadjuvant chemotherapy in patients with colorectal liver metastases) (31, 34). Considering that patients undergoing PVE/ALPPS experience two major interventions in a relatively short time, the patients may be at a high risk of malnutrition if additional calories and protein cannot be supplemented in time. These additional clinical conditions may further impair patients’ nutritional status. The higher incidence of sarcopenia also implies that the evaluation of body composition in these patients should be paid more attention to the perioperative assessment.

All included studies applied CT-based measurement for muscle mass assessment. This is reasonable given that CT is a commonly used imaging modality for the diagnosis and staging of patients with liver cancer. Furthermore, in the setting of PVE/ALPPS, CT is also widely applied for liver volumetry in pretreatment evaluation and to evaluate liver volume change after intervention. That is to say, the evaluation of body composition does not pose an extra burden for these patients. Dual-energy X-ray absorptiometry and bioelectrical impedance analysis are the other two commonly used methods for body composition measurement (35), but, to the best of our knowledge, they have not been employed in predicting liver remnant growth after surgery.

However, when analyzing the body composition, only limited details on CT imaging were provided in the included studies. Only two studies reported the imaging phase and just three studies described the slice thickness. It has been shown that these factors exert a considerable impact on the results of body composition analysis (36). In a study by Morsbach et al, the influence of contrast media and slice thickness on CT body composition segmentation was evaluated (37). They found that the skeletal muscle mass area, adipose tissue area, and muscle and fat attenuation (expressed in Hounsfield Units) showed a significant change after contrast media administration. There also was a significant effect on the area measurements (skeletal muscle mass area and adipose tissue area) when the slice thicknesses were adjusted. A systematic review summarized a group of CT-related factors which may affect the sarcopenia assessment (38). The CT parameters that according to the review can potentially affect the assessment included the use of contrast media, kilovoltage, CT manufacture and model, patient position, and slice thickness (38). Considering such many potential confounders, researchers need to bear them in mind when measuring body composition before transferring their results into clinical implementation. Besides, to make the findings reproducible and to increase the comparability among different studies, it also seems necessary to provide such information when reporting the body composition results.

Recent research has identified muscle quality, which can be determined by the infiltration of fat into muscle, as an independent prognostic factor in several types of cancer (39–41). In the present review, only one study adopted a composite index that combined skeletal muscle quantity and quality (named “muscularity”) (28), while the others only assessed skeletal muscle quantity. Theoretically, muscularity should have a better performance in the prediction of the clinical outcomes, including the FLR growth after PVE/ALPPS stage-1, but this needs to be confirmed by further research. Besides, as highlighted by the European Working Group on Sarcopenia in Older People 2 evaluation of muscle strength and physical performance is equivalent to the evaluation of muscle quantity and quality in the diagnosis of sarcopenia (20). Future research assessing the impact of sarcopenia on liver growth and the clinical outcomes in patients with cancer can also consider taking these components into account.

Even though an obvious heterogeneity was displayed in the included studies, all of them drew a similar conclusion that sarcopenia had a negative influence on liver growth after PVE/ALPPS stage-1. Furthermore, sarcopenia seemed to have an association with a higher risk of post-hepatectomy liver failure and lower surgical resection rate in the patient who underwent PVE/ALPPS, although the results were inconsistent in this review. Sarcopenia is also a risk factor for poor overall survival in patients with liver cancer (21). But the impact of sarcopenia on the overall survival of patients who undergo PVE/ALPPS remains unknown.

Tumor progression is another common reason for patients not being able to reach curative surgery after PVE. Its incidence is even greater than that of insufficient liver growth contributing to a “failed” PVE, 19% vs 11%, as reported in the international DRAGON trial (42). This may be partly due to the slow growth after PVE, approximately 4-8 weeks to induce an FLR growth of 12-38% (9, 43). During this long waiting interval, the tumor is likely to progress, leaving the patient not eligible for surgery anymore. Until now, only one study explored the influence of sarcopenia on the resectability in patients undergoing PVE. It showed that sarcopenia (defined as psoas muscle index < 500 mm^2^/m^2^) was a risk factor for unresectability (44). However, the sample size of that research was limited (only 88 patients). On the other hand, a meta-analysis that included 13 studies revealed that sarcopenia was also significantly associated with tumor recurrence (adjusted hazard ratio: 1.76) (21). Whether sarcopenia results in both impaired liver growth and increased tumor progression after PVE, and whether improvement of patient sarcopenia status can increase resectability and long-term prognosis are still unclear.

Even though all studies claimed that sarcopenia impaired liver growth, it is of note to point out that sarcopenia should only be considered as a cofactor that undermines FLR growth after PVE/ALPPS stage-1. To put it another way, other vital clinical variables also determine liver growth after PVE/ALPPS stage-1. Three of the included studies also detected initial FLR volume, total bilirubin level, and body mass index as independent risk factors for insufficient liver growth (28–30). As a prognostic factor, the initial FLR volume was also reported in previous studies (45–47). Other reported indicators include age (45, 48), embolic agent (49–51), segment IV embolization (52, 53), chemotherapy (45), and portal collaterals (54, 55). It is assumed that a combination of these risk factors may improve the predictive accuracy for the FLR growth after PVE/ALPPS stage-1.

There are some limitations in this study. This review was first limited by the small number of included studies, all with a limited sample size (median: 90). Also, there were no prospective studies and only one multicenter study. The lack of large prospective multicenter studies may undermine a convincing conclusion drawn from this systematic review. Second, due to the limited study number and methodological heterogeneity, it was not possible to perform a meta-analysis and synthesize the results to provide a pooled relative risk value of sarcopenia for poor FLR growth. Third, the limited number of studies and the research heterogeneity also made it difficult to identify the most accurate and reliable parameter for sarcopenia assessment, given that a variety of indices were used for muscle mass evaluation. As summarized in a review, as many as 14 methods are currently available for sarcopenia assessment (32). Nevertheless, an index combining skeletal muscle quantity and quality evaluation (for example, muscularity) seems more rational and effective. Future studies can be designed to compare these indices. Lastly, there seems to be a need to improve the research and reporting quality of studies on sarcopenia. For example, detailed information on CT imaging during body composition measurement is required to ensure a reproducible and reliable study.

## Conclusions

Research on the impact of sarcopenia on liver growth after PVE/ALPPS stage-1 is still in its initial stage. Based on currently available evidence, sarcopenia seems to have a high incidence in patients undergoing PVE/ALPPS and it may impair FLR growth. Its relationship with post-treatment complications, post-hepatectomy liver failure, and resection rate requires further comprehensive research.

## Data availability statement

The original contributions presented in the study are included in the article/**Supplementary Material**. Further inquiries can be directed to the corresponding author/s.

## Author contributions

Conceptualization, QW, and ZL. Methodology, QW and AW. Validation, AW, ZL, and TB. Formal analysis, QW, AW and ZL. Investigation, ZL. Data curation, AW, and ES; writing—original draft preparation, QW, and AW. Writing—review and editing, ES and TB. Visualization, QW. Supervision, ZL, and TB. Project administration, QW. Funding acquisition, QW.

## Funding

This research was funded by Medical Diagnostics Karolinska (grant number: 929646).

## Conflict of interest

The authors declare that the research was conducted in the absence of any commercial or financial relationships that could be construed as a potential conflict of interest.

## Publisher’s note

All claims expressed in this article are solely those of the authors and do not necessarily represent those of their affiliated organizations, or those of the publisher, the editors and the reviewers. Any product that may be evaluated in this article, or claim that may be made by its manufacturer, is not guaranteed or endorsed by the publisher.
